# The Defective and Dependent

**Published:** 1911-11-18

**Authors:** 


### THE DEFECTIVE AND DEPENDENT.

### II.—The Ameliorative Treatment of Defectives.

In briefly sketching the progress of Special Education of the various afflicted classes it will be convenient to begin with that of the blind and of the deaf, that of mentally and physically defective children having been undertaken at a later period.

### The Training of the Blind.

Until 1785 no systematic attempt to educate the blind except orally seems to have been made, but in that year Valentin Haüy, a native of Picardy, who had devised a plan for the tactual teaching of reading by means of alphabetic characters in relief, opened the first school for the blind in Paris. Later England took up the matter, and the earliest institution for the blind was founded at Liverpool in 1790. In 1793 Bristol and Edinburgh followed suit, and in 1799 the first London institution—that known as "The School for the Indigent Blind," long located in St. George's Road, Southwark, but now removed to Godalming—began its useful work. Other similar institutions were subsequently established throughout the kingdom, and in 1872 the Royal Normal College for the Blind at Upper Norwood came into existence through the benevolent exertions of Dr. Armitage. This establishment, under the able direction of Sir Francis Campbell, LL.D (himself a blind man), now comprises a training college for blind teachers of the blind, and a technical school for the older blind pupils, in addition to a well-equipped school for juniors, including a kindergarten. Up to 1879 little seems to have been attempted in the way of day-school instruction, but in that year the London School Board opened its first centre for the separate teaching of the blind, and six such day-schools now exist in London. A bequest by Henry Gardener of £300,000 became available in 1882 for the general furtherance of the education of the blind in England and Wales, and this fund has been helpful in promoting higher and technical training, especially in music. In 1889 the Royal Commission on the Blind, the Deaf and Dumb, etc., issued its report, and this led to the passing in 1893 of the Elementary Education (Blind and Deaf Children) Act, the principal provisions of which may be summarised as follows:—

1. Compulsory Education of blind children between 5 and 16 years of age.
2. The assignment to School Authorities of the duty of providing suitable instruction for blind pupils.
3. The provision of a special scale of Government Grants in aid of educating blind children in certified schools.

Under these conditions 38 certified schools—the majority residential—have become available under the Act, and about 2,300 blind children are thus provided for in England and Wales. The tendency in recent years has been to increase facilities for the industrial training of the older pupils.

### The Training of the Deaf.

The earliest scientific attempt at improving the condition of the deaf by enabling them to use articulate speech seems to have been made in 1692 by Dr. J. K. Amman, of Amsterdam, who may be regarded as the founder of the oral system, the details of his original method being described in his book entitled "Surdus Loquens." In 1760 the Abbé de l'Epée started in Paris a school for the deaf, whom he instructed on a system of manual sign language which he had elaborated. In 1788 the oral method was further developed by Heinicke, of Leipsic, and from that time came to be known as the "German System." The question of the superior utility of the manual or of the oral system has excited much controversy, but in this country oral teaching has of late years been more generally adopted. In 1792 the first English institution for training the Deaf was founded in the Old Kent Road, and has since developed into the important "Royal School for the Deaf" at Margate. The facilities offered by the Act of 1893 (which included deaf as well as blind), together with the establishment of two societies for the promotion in England of oral teaching, have given a considerable impetus to the education of the deaf, and there are now 48 certified schools—day and residential—for this class, with accommodation for over 4,000 pupils.

### The Training of the Defective.

The earliest systematic attempts at the training of the mentally defective date from the middle of the nineteenth century. At the Bicêtre Hospital in Paris Séguin commenced, in 1842, the special instruction of the imbecile children there, and with such good result that in 1846 he published a treatise setting forth his methods and showing how by education on physiological lines it had been possible materially to ameliorate the condition of many of his pupils. About the same time Guggenbühl established on the Abendberg, a mountain station above Interlachen, an institution for Cretins where some remarkable results were achieved by judicious hygienic and educational measures. Saegert, Director of the Asylum for Deaf Mutes in Berlin, brought to the notice of the authorities the success he had had in dealing with children whose speech was defective as a consequence not of deafness but of mental weakness, and in 1845 he established a separate private establishment for such cases. In Great Britain the first training school for imbeciles was opened in 1846 at Bath, and in 1847 came the establishment at Highgate of the charity which afterwards developed into the large institutions at Earlswood and Colchester, and set an example subsequently followed in other parts of the kingdom. These institutions, originally known as Asylums for Idiots and Imbeciles, demonstrated what could be done by persevering training in cases previously considered hopeless.

